# Salvage surgery for patients with residual/persistent diseases after improper or insufficient treatment of oral squamous cell carcinoma: can we rectify these mistakes?

**DOI:** 10.1186/s12885-021-08600-2

**Published:** 2021-07-31

**Authors:** Yue He, Zhonglong Liu, Surui Sheng, Weijin Gao, Xiao Tang, Xiaoguang Li, Chunyue Ma

**Affiliations:** 1 Department of Oral & Maxillofacial – Head & Neck Oncology, 9th People’s Hospital, Shanghai Jiao Tong University School of Medicine, Key Laboratory of Stomatology, No. 639, Zhi Zao Ju Road, Shanghai, 200011 Shanghai China; 2 Department of Oral and Maxillofacial Surgery, The First Affiliated Hospital of Wenzhou Medical University, Wenzhou, 325000 Zhejiang China

**Keywords:** Salvage surgery, Oral squamous cell carcinoma, Persistent disease, Recurrent disease, Surgical margin, Survival, Management

## Abstract

**Background:**

Patterns of failure after treatment of oral and squamous cell carcinomas (OSCC) are diversified, with recurrences being one of the common causes. A special group of patients are sometimes encountered in the outpatient clinic for improper or insufficient initial treatment with reports of positive margins, implying residual/persistent diseases. The question of whether these patients can be surgically salvaged remain unanswered.

**Methods:**

A retrospective study was performed between January 2013 and December 2017 for patients with residual or rapid recurrent (within 3 months) OSCCs, who received salvage surgeries in our institution. The patients with residual/persistent OSCCs were those with microscopic or macroscopic positive surgical margins, while those with rapid recurrent OSCCs were those with close or negative margins, but unabated painful symptoms right after treatment. Both clinicopathological and prognostic variables were analyzed. The focus was also directed towards lessons for possible initial mistakes, resulting in these residual/persistent diseases.

**Results:**

Of 103 patients, 68 (66%) were men, with mean age of 56.3 years. The overall survival reached 60.2%. Regarding the primary OSCC status, most of our patients (*n* = 75, 72.8%) were diagnosed with ycT2–3 stages. Besides, most patients were found with macroscopic residual diseases (52.4%) before our salvage surgery. The sizes of the residual/persistent OSCCs were generally under 4 cm (87.3%) with minimally residual in 21 (20.4%). Among all the variables, primary T stage (*p* = 0.003), and residual lesion size (*p* < 0.001) were significantly associated with the prognosis in multivariate analysis. Though the causes for the initial surgical failure were multifactorial, most were stemmed from poor planning and unstandardized execution.

**Conclusions:**

Cases with residual/persistent OSCCs were mostly due to mistakes which could have been avoided under well-round treatment plans and careful surgical practice. Salvage surgery for cases with smaller residual/persistent OSCCs is still feasible with acceptable outcomes.

**Supplementary Information:**

The online version contains supplementary material available at 10.1186/s12885-021-08600-2.

## Background

Primary standard of care for oral squamous cell carcinoma (OSCC) patients remains surgical, with or without adjuvant therapies [1]. Though advancements of techniques and regimens have resulted in cure in about 60% of patients, failure of such treatment is still common [1, 2]. Most of authors tended to owe various patterns of treatment failures to recurrences or/and distant metastases [3, 4], based on disease subsites. However, the vague definition of “recurrences” in most reports have confused two groups of patients, residual/persistent and recurrent, who were drastically distinct in terms of clinical characteristics and prognoses, as patients with residual diseases were mostly those with initial positive margins [5]. Even within the so-called “recurrent” patients, some were however actually suffered from unabating/persistent painful symptoms right after initial resections, indicative of vastly inappropriate treatment, though with questionable prior reports of “negative” margins [6, 7]. From our perspective, despite the initial curative intent, most cases with positive margins or persistent symptoms (confirmed by biopsies) should be regarded as “residual/persistent”, rather than “recurrent” OSCCs, on account of their insufficient prior treatment, and the unexpected survival loss accompanying abrupt changes of pre-decided treatment plans [8]. Although some recent studies started to recognize such patient subgroups as with rather “residual/persistent” diseases for their particular treatment history, most still simply regarded these patterns of failure as “relapsed” lesions [2, 8, 9]. The philosophy behind such insistence was primarily established on the sole concern with margin positivity, irrespective of several other clinicopathologic factors, such as history of adjuvant therapy (blurring true margins), accuracy of margin assessment (specimen-driven or tumor-bed driven), number of margins, times of secondary negative margins (i.e. initially positive margins), tumor sizes, infiltrative depths and extirpation routes (surgical exposure) [2, 10–13]. Therefore, without scrutinizing prior surgical procedures, in our opinion, it is probably a bit far-fetched to assigning local surgical failures of heterogenous causes into one single category of recurrence, especially for patients who “recur” rapidly within just weeks after primary surgical resections. According to some, such blame of residual/persistent OSCC was still mostly directed towards so-called aggressive tumor biology, making the disaster of rapid re-growing of OSCC seem destined [11, 14]. On the other hand, there was also a prevailing notion that these residual/persistent diseases primarily reflected inadequacy of resection due to strong associations between incomplete tumor extirpation and prompt tumor reappearances [2, 8]. Unfortunately, most studies barely scratched the surfaces on the causes behind such clinical mismanagement. Apart from these, most of these residual/persistent cases will seek possible immediate retreatment or consultation in other larger regional or national cancer centers, especially for chances of surgical re-resections. However, there were limited data or evidences available describing the treatment and prognosis for such subgroups of patients.

Thus, in light of the situations, we try to share our experiences highlighting the causes in these OSCC patients with residual /persistent diseases. The other aim of our study was to investigate whether immediate salvage surgery (SS)-based efforts can rectify the mistakes of undertreatment in these patients.

## Methods

We performed a retrospective chart review of all the patients who received SS for rapid recurrent OSCCs from January 2013 to December 2017 in our institution, a national referral center for head and neck cancer treatment. The collection and analyses of the data was approved by the institution’s ethics committee. The reference number is SH9H-2020-T300–1. All the patients included in this study gave their written consent to participate in this study. Besides, written consent of publications were obtained for all the patients in this study for use of their data and images relevant to this study.

The detailed inclusion (eligibility) criteria were as follows: 1) patients who had the histories of surgical treatment for primary OSCCs in other institutions; 2) those with residual diseases confirmed by reports for positive margins, or those with persistent diseases (i.e. negative margins but encountering rapid recurrences within 3 months); 3) patients consent to immediate SS and received re-treatment within 3 months after previous treatment; 4) those with postoperative (after SS) pathological confirmation of residual/persistent OSCCs. Patients included should meet all the above inclusion criteria. Besides, the exclusion criteria were as follows: 1) patients who received non-surgical treatment only; 2) those with distant metastases; 3) those with unknown information regarding primary treatment.

Demographic data, medical histories, comorbidities, OSCC characteristics, and pretreatment status were collected. Emphasis was given towards the patterns of the initial treatment, with possible causes for previous undertreatment failures. Clinical stages regarding the primary OSCCs were based on American Joint Committee on Cancer (AJCC), 8th edition [15]. Besides, pathological information about depth of invasion (DOI) and extra-nodal extension (ENE) were sent and reviewed again by the Department of Pathology for those receiving primary treatment before 2018. Other clinicopathological parameters regarding the number of positive margins, sizes of residual lesions, midline involvement, differentiation, neurovascular invasion, mandibular/maxillary bone invasions were also collected.

The possible causes for initial surgical failure were analyzed with a focus on the referral reports. The communication records (with the initial treatment clinics), and the patients’ counseling records were also found in the outpatient clinical database. Specific causes were summarized after reviewing the information.

Based on the varied admission status to our institution, all the included patients were firstly classified into three categories: patients with microscopic residual diseases (MiRDs); with macroscopic residual diseases (MaRDs) or with rapid recurrent (persistent) diseases (RRDs). MiRDs were defined as those with prior reports of initial positive margins, and suspected residual diseases via physical examination (indeterminant post-treatment fibrosis/infection or recurrences) yet unconfirmed imaging (invisible or indeterminant), while MaRDs were those with gross (both physically palpable and radiographically visible) residual diseases and initial positive margins, while RRDs were defined as those with initial false negative margins but visible persistent diseases right after prior operations (< 3 months). Generally speaking, patients in MiRDs group were mostly with minimally residual lesions or residual lesions less than 2 cm in size, while those in MaRDs group were those with larger residual lesion sizes. Usually, the patients in MiRDs group were most anxious about the prior pathologic results and sought for retreatment immediately after the undertreatment in other institutions. We would firstly perform preoperative or intraoperative incisional biopsies in suspected areas, followed by radical re-resections with enlarged peripheral margins when the suspicions were confirmed. Apart from that subclassification, as far as the involved oral subsites were concerned, the studied cohort was also divided into three groups: with local residual, regional (cervical) residual, and both locoregional residual diseases. In consideration of the distinct residual/persistent tumor sizes, the extent of SS was tentatively graded according to the re-resections and reconstructions: 1 for simple re-resections, or simple re-neck dissections followed by direct wound closure or local flaps coverage; 2 for radical re-resections with reconstructions with pedicled pectoralis major myocutaneous flaps (PMMF) or free flaps; 3 for SS involving craniofacial resection (skull base), carotid artery resection, total glossectomy, total maxillectomy with orbital exenteration, or hemi-mandibulectomy, with free-flap reconstructive procedures.

In order to analyze the efficacy of our immediate salvage treatment, these patients were also classified into two groups based on different presurgical treatment: patients received upfront SS (SS group); and those underwent adjuvant treatment regimens first, followed by SS (AT-SS group). AT-SS treatment was mostly offered to patients with larger residual/persistent lesion sizes, poorer pathologic differentiation and other adverse clinicopathologic features. The major intention was for possible tumor size reduction. The SS complications were recorded as well. In addition, various adjuvant treatment modalities were applied to these patients after multidisciplinary case-based discussions. SS without adjuvant therapies were mostly applied to patients in MiRD group with small or minimal residual lesions, or those with serious comorbidities who were unfit for such combinations. Radiotherapy or chemoradiation was routinely administered to these patients, while the latter was more frequently applied to those of younger ages, with larger primary or residual tumor sizes, or with adverse pathologic features, such as PNI or bone invasion. The supplement of targeted therapies to these conventional adjuvant therapies was determined by multivarious factors, such as target protein expression, age, comorbidities and affordability due to high costs. For sake of statistical analysis, the postoperative adjuvant treatment (after SS) was further summarized into five categories: none, radiotherapy, chemoradiation, radiotherapy with targeted therapy, and chemoradiation with targeted therapy. None of the patients received simple postoperative chemotherapy with/without targeted therapy. The targeted drugs included epithelial growth factor receptor inhibitor (Nimotuzumab or Cetuximab), and vascular endothelial growth factor receptor inhibitor (Apatinib). However, due to the unavailability of programmed death-1 (PD-1) related pembrolizumab at that time, immunotherapies were not applied in any of these patients.

Overall survival (OS) time was calculated as the time from the start of SS to death/last outpatient visit in months. Salvage outcomes were recorded and compared between these patients with residual/persistent OSCCs. The Chi-square test and Fisher exact test were used to compare categorical variables. Univariate log-tank test was adopted to analyze survival time-dependent variables. Subsequently, Cox regression analysis was carried out on the variables that achieved univariate statistical significance. All statistical analyses were conducted via SPSS 21 for Windows (IBM Corp., Armonk, NY).

## Results

### Demographic information

During the 5-year interval (from 2013 to 2017), a total number of 1761 patients with recurrent malignancies had received SS in our institution, according to the chart database. Within these patients, 103 (5.84%) met our inclusion criteria. Of these, 68 (66%) were men and the rest were women (*n* = 35, 34%); The average age reached 56.3 years, of whom 42.7% were smokers. Most patients (*n* = 36, 35.0%) were with initial diagnoses of tongue cancers, followed by buccal (20.4%), lower gingival (20.4%) and floor-of-mouth (20.4%). The mean follow-up reached 31.1 months (range, 4–65 months). The detailed demographics were summarized in Table 1. Representative cases were presented in Supplementary Figs. 1 to 4.

**Table 1:** Demographic and clinicopathological characteristics for initial treatment

Characteristics	Number (%)	Overall survival (%)	***p (***Log-rank)
**Sex**			
Male	68(66.0)	67.6	0.046
Female	35(34.0)	48.6	
**Age**			
< 60	60(58.3)	60.0	0.744
≥ 60	43(41.7)	60.0	
**Comorbidity**			
Cardiovascular diseases	20(19.4)	70.0	0.459
Diabetes	7(6.8)	57.1	
Others	4(3.9)	100.0	
Combinations	12(11.7)	66.7	
None	60(58.3)	55.0	
**Site of primary OSCC**			
Tongue	36(35.0)	61.1	0.600
Floor of mouth	13(12.6)	76.9	
Bucca	21(20.4)	70.0	
Lower gingiva	21(20.4)	47.6	
Upper gingiva	8(7.8)	62.5	
Hard palate	4(3.9)	50.0	
**Primary T stage**			
T1	2(2.0)	100.0	0.011
T2	27(26.2)	74.1	
T3	48(46.6)	64.6	
T4	26(25.2)	38.5	
**Primary N grade**			
N0	64(62.1)	62.5	0.344
N1	19(18.4)	68.4	
N2	10(9.7)	40.0	
N3	10(9.7)	60.0	
**Primary stage**^**#**^			
Early stage	23(22.3)	65.2 (56.2)	0.680
Late stage	80(77.7)	60.0 (43.4)	
**DOI of primary OSCC**			
> 10 mm	45(43.7)	68.9	0.092
≤ 10 mm	58(56.3)	55.2	
**ENE of primary OSCC**			
With ENE	10(9.7)	61.3	0.757
Without ENE	93(90.3)	60.0	
**Primary histological grade**			
Well differentiated	16(15.5)	68.8	0.747
Moderately differentiated	65(63.1)	60.0	
Poorly differentiated	22(21.4)	59.1	
**Neck dissection for primary OSCC**			
Bilateral neck dissections	8(7.8)	65.9	0.116
Ipsilateral neck dissection	51(49.5)	60.8	
None	44(42.7)	37.5	
**Margin status after primary surgery**			
Positive	80(77.7)	60.0	0.711
Negative	23(22.3)	65.2	
Number of positive margins^*^			
1	54(67.5)	63.0	0.711
2	25(31.3)	52.0	
3	1(1.3)	100.0	
Positive deep margin^*^			
Yes	37(46.3)	56.8	0.714
No	43(53.8)	62.8	
**Primary reconstruction**			
Primary closure	18(17.5)	66.7	0.117
Local flap or skin graft	57(55.3)	66.7	
Free flap and PMMF	28(27.2)	46.4	
**Neoadjuvant therapy for primary OSCC**			
Yes	14(13.6)	35.7	0.006
No	89(86.4)	65.2	
**Ceased adjuvant therapy after primary surgery**			
No adjuvant therapy	98(95.1)	60.2	0.432
Ceased adjuvant therapy	5(4.9)	80.0	

*: Only patients with positive margins were included

^#:^The number in the parenthese represented the 5-year salvage rates of the patients with due follow-up

### Initial treatment and possible causes for failures

With regards to the primary disease status, most patients (*n* = 74, 71.8%) in our series were diagnosed with primary T3-T4 stages. Within these, DOI > 10 mm was confirmed in 45 (43.7%) patients after pathological review, resulting in the increased 21 (20.4%) cases of T3 stages. The patterns for primary N stages were different, with merely 20 (19.4%) patients with higher nodal metastasis grades (N2–3). The pathological grades of the primary OSCCs were as follows: well differentiated grades in 16 (15.5%) cases, and poorly differentiated ones in 22 (21.4%). Neoadjuvant chemotherapy was applied in 14 (13.6%) patients, while most received upfront primary surgery. As for the extent of the initial surgery, a total number of 59 (57.3%) received ipsilateral or bilateral neck dissections. When comparing the surgical procedures, direct wound closure and local flaps were mostly frequently used for defect coverages (72.8%). The important postoperative reports for surgical margins revealed that 77.7% (*n* = 80) were positive, within whom an astounding number of 53 patients (51.5%) were without any reports of frozen intraoperative margins according to the referral information. Judging from the initial reports, the primary resections were quite arbitrary, with 26 cases with two or more positive margins. Unfortunately, of these OSCC patients, a large proportion (*n* = 37, 35.9%) were with insufficient deep margins, implying ill-considered surgical decisions or incomplete resections. Within the whole group, 23 (22.3%) patients were with reports of “negative” margins while they still suffered from persistent symptoms (mostly pain) after prior surgical treatment. A closer inspection of these initial “negative” margins revealed that 14 (13.6%) were suspected for close margins (< 5 mm), while 9 (8.7%) were found with intraoperative re-resections due to firstly positive-margin reports. All these “negative” margins were later doubted due to the confirmed biopsies prior to SS. The initial radiotherapy was administered to 5 patients, yet all ceased and resort to retreatment in our institution. Thus, most patients in this study were radiation-naïve. (Table 1).

### Referral status and initial failure analysis

Within these patients with unsuccessful prior treatment, most (*n* = 78, 75.7%) were referred by other institutions in the first place, while some referral requests were initiated by the patients themselves. After communication with all the transfer-applying doctors, most of the referred patients (74, 71.8%) received initial surgical treatment in institutions of low-volume OSCC cases loads (< 50 cases per year), while others were from institutions of high-volume OSCC cases loads. Nineteen (18.4%) patients were of elderly ages over 70, while comorbidities were found in 41.7% (*n* = 43) of the entire cohort. Interesting, we also found insufficient surgical margin information (≤3 margins taken/patient) in almost half (45.6%) of the referral reports, indicative of inadequate margin analyses. Besides, the clinical diagnoses were mistaken for other pathologies in 17 (16.5%) patients, while even 23.3% patient received initial operations without confirmations by preoperative biopsies. Within those who received preoperative biopsies, improper delays (> 2 months) between biopsy and surgery were found in 16 patients (15.5%), due either to patients’ or iatrogenic reasons. Interesting, the counselling records with revealed that a striking number (*n* = 41, 39.8%) of patients in this study were reluctant to receive radical tumor resections with free-flap reconstructions in the initial treatment settings. Besides, from a surgical standpoint, treatment design loopholes were also plentiful in these cases, with mostly insufficient margins of depth (35.9%), mismatch between lesion sizes and resection/reconstruction methods (31.1%). Unstandardized operative practices were suspected as well for residual lymph nodes found in the cervical basin (*n* = 24, 23.3%) after initial neck dissections. On the other hand, non-en-bloc (non-continuity) resections were applied in T4 cases (*n* = 11, 10.7%) involving tongue, floor of mouth, low gingiva, resulting in possible residual lesions in the middle zones. *(*Table 2*).*

**Table 2:** Referral status and possible causes for residual/persistent OSCCs

Referral status and possible causes	Number (%)
**Referral status**	
Institutional referral	78 (75.7)
Patient’s decision	25 (24.3)
**Primary treatment center**	
with low-volume oral cancer cases	74 (71.8)
with high-volume oral cancer cases	29 (28.2)
**Surgeon’s expertise**	
Junior consultant	22 (21.4)
Senior consultant	69 (67.0)
Surgeons of nonrelated specialty	12 (11.7)
**Age of patients**	
<70	84 (81.6)
≥ 70	19 (18.4)
**Comorbidities**	
Yes	43 (41.7)
No	60 (58.3)
**Reports of intraoperative frozen section**	
Yes	50 (48.5)
No	53 (51.5)
**Report completeness for primary margins**	
≤ 3 margins	47 (45.6)
>3 margins	56 (54.4)
**Clinical stage**	
Early stage	23 (22.3)
Late stage	80 (77.7)
**Clinical diagnosis before primary surgery**	
Correct	86 (83.5)
Wrong	17 (16.5)
**Biopsy before primary surgery**	
Yes	79 (76.7)
No	24 (23.3)
Time Lag between outpatient biopsy to primary admission*	
≤ 2 month	63 (79.8)
> 2 month	16 (20.2)
**Patient’s initial reluctancy to radical resection/reconstruction**	
Yes	41 (39.8)
No	62 (60.2)
**Treatment design mistakes**^**&**^	
Flawed access for advanced cases	21 (20.4)
Undertreatment regarding tumor depths	37 (35.9)
Mismatch between imaging sizes and resection methods	32 (31.1)
None of the above	41 (39.8)
**Unstandardized operative implementations**^**&**^	
Residual positive lymph node in operated cervical basin	24 (23.3)
Non-enbloc resection for advanced lesions	11 (10.7)
None of the above	78 (75.7)

^*^: Only patients with biopsies before primary surgeries were included

&: These different mistakes might overlap in the primary treatment of the same patients

### Clinicopathologic data for residual/persistent OSCCs

According to the previously mentioned classification, most patients in our study were in the MaRD group. In terms of size, most of the residual OSCCs (*n* = 69, 67.0%) were not larger than 4 cm, suggestive of the curable local conditions. Within these, 45.7% were with gross (> 2 cm) residual lesions. Upper and lower vital-structure (skull base, orbit, carotid artery) involvement were found in approximately 13% of the cases, for whom SS was even more challenging. Compared with initial pathologic reports in primary treatment, pathologic differentiation upgrades were found in 23 (22.3%) patients, indicative of the increased aggressiveness of residual/persistent OSCC. Besides, neurovascular invasions were also confirmed in 19.4% of the cases. As for salvage treatment, eighty-five (82.5%) cases received upfront SS, while others (*n* = 18, 17.5%) were in the AT-SS group. Within those receiving AT-SS sequential treatment (n = 18), 16 patients were also in the MaRD group, while the other 2 in the RRD one. The mean residual OSCC size reached 5.1 cm, with poorly differentiation in 10 cases and with bone invasion in 12. The extent of SS was more extensive than the initial treatment, with 78.5% (*n* = 84) receiving radical resections and flap reconstructions. Within these patients, complicated wide-excision surgery, such as craniofacial skull base surgery, total glossectomy, carotid artery resection, total maxillectomy or hemi-glossectomy were not rare (25.2%). In terms of internal or common carotid sacrifice, a closer inspection of our data revealed that 3 (2.9%) patients underwent common or internal carotid artery resection with mean arterial stump pressure over 50 mmHg. All these 3 cases with carotid artery sacrifices went on to receive postoperative sequential chemoradiation with Nimotuzumab treatment. Unfortunately, none of these patients survived during the follow-up. Despite our SS, post-salvage margin reports still revealed positive margins in 4 (3.9%) cases, of whom most were with larger (> 4 cm) residual OSCCs, or lesions, extending near or through vital structures. In addition, 37 patients experienced complications in postoperative settings. Most were minor wound infections or lung infections. In addition to SS, adjuvant therapies based on radiotherapy or chemoradiation, were offered to most cases (*n* = 84, 81.6%), while targeted therapies were to 17 (16.5%) (Table 3).

**Table 3:** Clinicopathologic information about residual/persistent OSCCs

Characteristics	n (%)	OS (%)	***p*** (Log-rank)
**Residual subgroup**			
MiRD group	26(25.2)	84.6	0.005
MaRD group	54(52.4)	48.1	
RRD group	23(22.3)	65.2	
**Residual OSCC location**			
Local	79(76.7)	60.8	0.950
Regional	12(11.7)	66.7	
Locoregional	12(11.7)	58.3	
Size of rOSCC^*^			
Minimal residual	21(20.4)	81.0	< 0.001
≤ 2 cm	35(34.0)	71.4	
2-4 cm	34(33.0)	55.9	
> 4 cm	13(12.7)	15.4	
**rOSCC involving vital structures**^**#**^			
Yes	13(12.6)	38.5	0.049
No	90(87.4)	64.4	
**Differentiation for rOSCC**			
Well differentiated	9(8.7)	66.7	0.857
Moderately differentiated	60(58.3)	61.7	
Poorly differentiated	34(33.0)	58.8	
**Pathological upgrade for rOSCC**			
Yes	23(22.3)	69.6	0.583
No	80(77.7)	58.8	
**Neurovascular invasion for rOSCC**			
Yes	20(19.4)	70.0	0.583
No	83(80.6)	59.0	
**Bone invasion for rOSCC**			
Yes	31(30.1)	45.2	0.023
No	72(70.0)	70.0	
**Margin status after salvage surgery**			
Positive	4(3.9)	0.0	< 0.001
Negative	99(96.1)	63.6	
**Complication after salvage surgery**			
Yes	37(35.9)	56.8	0.488
No	66(64.1)	63.6	
**Detailed complications**			
Wound dehiscence or infection	14(37.8)	57.1	0.436
Flap crisis or failure	4(10.8)	25.0	
Lung infection	12(32.4)	58.3	
Chyle leak	1(2.7)	100.0	
Deep venous thrombosis	2(5.4)	100.0	
Hematoma	1(2.7)	100.0	
Others	3(8.1)	33.3	
**Salvage treatment combinations**			
Upfront salvage surgery	85(82.5)	64.7	0.033
Adjuvant therapy followed by salvage surgery	18(17.5)	44.4	
**Salvage resection and reconstruction**			
Simple resection with direct closure or local flap	19(18.4)	89.5	0.001
Radical resection with free flap or PMMF coverage	58(56.3)	62.1	
Craniofacial surgery, total glossectomy, etc	26(25.2)	38.5	
**Adjuvant therapy**			
Radiotherapy	43(51.2)	58.1	0.156
Chemoradiation	25(29.8)	60.0	
Radiotherapy with targeted therapy	10(11.9)	50.0	
Chemoradiation with targeted therapy	6(7.1)	33.3	
None	19(18.4)	84.2	

* Sizes of residual OSCCs (rOSCC) were determined by pathological reports, with minimal residual lesions signifying lesions smaller than 1 cm

#: Vital structures: Carotid artery, skull base or higher, glottic, hypopharynx, larynx

The correlations between the parameters regarding initial treatment and residual retreatment were analyzed (Table 4). Both primary T stage (*p* < 0.001) and neoadjuvant therapy (*p* = 0.024) were found to be related to residual/persistent lesion size. Primary T stage was also correlated with residual subgroup (*p* < 0.001), and vital-structure involvement (0 = 0.017).

**Table 4:** Correlations between clinicopathological characteristics between initial and residual/persistent OSCCs

Initial treatment	Residual retreatment	***p*** values
**Primary T stage**	**Residual subgroup**	**< 0.001**
	**Size of residual OSCC**	**< 0.001**
	**rOSCC involving vital structures**	**0.017**
	**Salvage treatment combinations**	**< 0.001**
	**Salvage resection and reconstruction**	**0.001**
**Neoadjuvant therapy for primary OSCC**	**Size of residual OSCC**	**0.024**
	**Salvage treatment combinations**	**0.002**
	**Salvage resection and reconstruction**	**0.011**

### Survival outcomes and statistical analyses

**Fig. 1:**
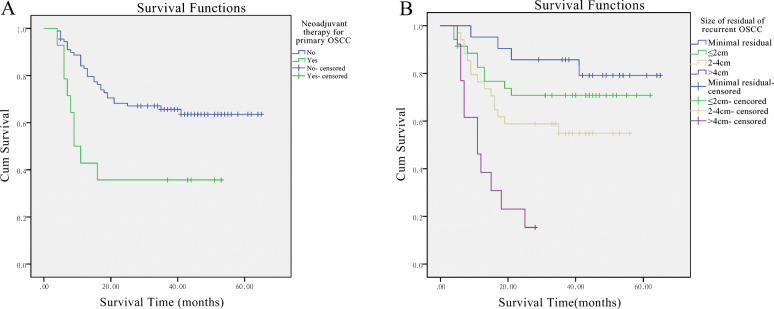
The Kaplan-Meier curves for the significant variables in Cox multivariate analyses. A. Primary T stage; B. Residual OSCC size

The OS rate (main outcome) reached 60.2%, with 41 deaths within the whole group, while the 5-year salvage rate (*n* = 69) dropped to barely 49.3% when taking into the 5-year criteria duration of follow-up, with 56.2% (*n* = 16) for early-stage cases and 43.4% (*n* = 53) for late-stage ones (Table 1). When it comes to the specific death causes, locoregional re-recurrences were found in 26 (25.2%) cases, while both recurrences and distant metastases in 11 (10.7%), representing the two major reasons for our salvage failures. The univariate log-rank analyses of the initial treatment data revealed that sex (*p* = 0.046), primary T stage (*p* = 0.011) and neoadjuvant therapies (0.006) were related to the patients’ prognosis. As for the residual/persistent OSCC data, the univariate analyses showed significances in residual/persistent subgroups (*p* = 0.005), size of the residual OSCC (*p* < 0.001), vital-structure involvement (*p* = 0.049), bone invasion (*p* = 0.023), salvage margin status (*P* < 0.001), salvage treatment combinations (0.033) and salvage resection and reconstruction extent (0.001). Among all the variables, both primary T stage (*p* = 0.003), and residual lesion size (p < 0.001) were significantly associated with OS, based on the final Cox multivariate analysis (Table 5*,* Fig. 1).

**Table 5:** Cox multivariate analysis of SS for patients with residual/persistent OSCCs

Parameter	*p*	HR^a^	95.0% CI^a^	
Primary T stage	0.003	1.96	1.262	3.044
Size of residual OSCC^b^	< 0.001			
≤ 2 cm	< 0.001	0.112	0.035	0.357
2-4 cm	< 0.001	0.201	0.084	0.480
> 4 cm	0.006	0.328	0.149	0.723

^a^*HR* Hazard Ratio, *CI* Confidence Interval

^b^The analyses was based on the reference of minimal-residual group

## Discussion

It is well known that the best opportunity to cure patients with OSCC is through the delivery of fast and appropriate therapy at first presentations [7, 15]. Theoretically speaking, management of “recurrence” after prior treatment is a challenging clinical situation, with decreased chances of cure by retreatment [16, 17]. Although there is no standard criteria or consensus of a “true recurrent” OSCC, most still consider “recurrences” as those with similar pathological profiling, involving nearby anatomic structures (< 3 cm) and within 3 years of follow-up [14]. In literature, such “recurrences” were only divided by years, as either rapid or late recurrences, irrespective of detailed previous primary treatment [14, 17–19]. As far as we are concerned, initial treatment modalities, prior surgical margin and postsurgical symptom (pain) should all be taken into consideration when differentiating true “recurrent” and “residual/persistent” OSCCs, as some “recurrences” were in fact residual/persistent lesions (with evidence of residual diseases or without intermittent remission of symptoms) [13]. We figure that these OSCCs become residual due more to improper initial treatment or insufficient resections, rather than to oncological aggressiveness of OSCCs. Determining the optimal retreatment regimens for this special group is very important, as most patients are extremely anxious about the likelihood of rapid and curative salvage re-resections [14]. According to our referral/admission analysis, the report of positive margins, along with the unrelieved painful symptoms, always encroached on the retreatment confidence in the primary treatment centers, given the fact that a high proportion (24.3%) of referrals were actually requested by patients. As occasionally encountered with these referrals, we tried to answer the question of whether these patients with residual/persistent OSCCs could still be rescued with SS-based treatment, as controversy for such decisions still exists [11, 19, 20].

Such residual/persistent OSCC problems were caused by several factors, which however has long been under-evaluated. To a large extent, initial (primary) treatment status will negatively influence the survival outcomes [20]. Firstly, the factors of surgeons should not be downplayed. According to the referral reports and patients’ statements, the initial surgical treatment was carried out in some patients with unproven preoperative biopsies, which violated the principles of National Comprehensive Cancer Network (NCCN) guidelines [21]. Such condition was mostly due to surgeons’ false biopsy practices or lack of experiences for early OSCC diagnoses. Besides, sometimes the variety of clinical presentations of OSCC, and possible concurrence of other premalignant oral lesions will also confuse or delay the early clinical diagnosis [22]. It has been widely acknowledged that an early and correct diagnosis is of utmost importance in reducing pretreatment intervals, providing standardized care and reducing mortality [22–24]. Mistakes of wrong or delayed OSCC diagnosis can be avoided with heighten awareness, as well as with extended use of novel techniques. Recent studies have also explored the possibility of having a set of biomarkers for assessment of suspected lesions, or differentiating between these benign and malignant oral lesions [25–27]. Despite high heterogeneity of these researches, it is interesting to find protein alterations in different genomic proteins during OSCC formation or development for possible early surgical interventions [26–28]. Apart from tissue genomic examinations, several non-invasive imaging OSCC diagnostic aids, such as fluorescence detection, can also be utilized to overcome the limits of routine oral examination. While not as informative as biopsy, these methods can aid in early identification of malignant transformation [29]. In addition, from a baseline diagnostic perspective, single or multiple incisional biopsies are also required for large and non-homogenous lesions to confirm the OSCC diagnosis preoperatively [18, 30]. The other mistake was the surgical completeness [31]. Mismatch between primary OSCC stages and resection/reconstructive methods were abundant in our series, as some locally advanced lesions (*n* = 32, 31.3%) were even resected and reconstructed with direct closure or local flaps. Thus, the radicality of initial treatment was seriously questioned in these cases. In addition, a fairly large number of the cases in our study were with initial positive deep margins, implying possible flawed intraoperative resection regarding the tumor depth, which will finally compromise the treatment efficacies [32–34]. Due to the terrible margin status in most of the patients, we advocate that en-bloc, or even compartment surgeries should be strongly recommended to ensure margin safety, particularly for adequate deep margins in advanced primary cases [35, 36]. Interestingly, even in some cases with primary early-stage OSCCs, residual lesions were still found in the tumor basins. We figured that such iatrogenic mistakes, which could have been avoided, were mostly due to unprepared preoperative surgical plans. For example, for cases with tongue cancers, the para-glossal resections should not be overly conversed for lingering fear of oro-cervical communications. The removal of sublingual gland and floor of mouth mucosa should also be advocated for a clear middle-zone eradication [37]. For cases with buccal cancers, especially those in the anteromedial buccal subsites, thorough-and-thorough resections should be attempted despite possible cosmetic disfigurement. For retromolar and lower buccal lesions, the resections of medial, sometimes lateral pterygoid muscles, marginal medial mandibulectomy should always be highlighted in those with clinically presentations of seemingly “early-stage” diseases, but true invasive fronts regarding the tumor depths [38]. Anatomically speaking, these parapharyngeal structures are adjacent, or in direct connection with the oral epithelial tissues, where improper surgical practice will result in positive margins [39]. Considering the treatment outcomes of these residual lesions, it is better to “err on the safe side” for extending the margins a bit wider, and to prepare intraoperative flap reconstructions, especially for some clinically ycT2–3 cases [31, 32]. Besides, the existence of cervical residual OSCCs were, in our opinion, partly due to unstandardized or improper resections or neck dissections, and to higher primary N grades [16, 40]. We consent to the recent Clinical Practice Guideline issued by American Society of Clinical Oncology for establishing preliminary recommendations on the criteria of a high-quality neck dissection [16]. The anatomic hallmarks, levels and lest number of nodal specimens should also be emphasized for the best practice of primary surgical care for OSCC patients.

Apart from the surgical problems, as reflected in Table 2, other clinical factors should also be cautiously evaluated for avoiding treatment malpractice. Firstly, as is reflected in our series, 41.7% of the cases were with comorbidities, which might cause hesitations of aggressive surgical treatment from the patients’ and doctors’ perspectives [41]. Besides, the competencies of surgeons for such OSCC treatment should be assessed [42], as 33.1% of the patients in our study received their initial treatment from junior consultants, or even surgeons from other non-relating specialties. Besides, patients who received surgical treatment from low-volume peripheral institutions tend to have improper or low-quality practice in our series, with more chances of positive margins and lower likelihood of providing care adherent to guidelines [43, 44]. However, such view was refuted by Eskander for the conflicting evidence comparing the quality of care between high- and low-volume institutions [45]. For us, the ample experiences of treating OSCCs on a regular basis made difference between institutions and surgeons. In addition, the adverse survival relationships of “delays between biopsies and treatment” was consistent with the reports of others [46]. Due to such varied negligences in primary treatment, we call for strictly adhering to the treatment and diagnosis guidelines otherwise it may cause tremendous disaster to the patients. Conversely, improper management for OSCC will cause locoregional failure and even death [11–13]. The preoperative plan including surgical approach, reconstructive method and adjuvant therapy of oral cancer needs a multidisciplinary team to achieve the best clinical outcomes. A qualified and experienced surgical oncologist is prerequisite for the ultimate success of treatment. As revealed in our study, undertreatment from inexperienced surgical oncologist will lead to a dismal outcome and is not acceptable in the current standard of care.

For the treatment of resectable residual/persistent diseases, there were still unsettled controversies about the role and outcomes of SS, with vastly conflicting survival outcomes ranging from 8.3 to 62.5% [6, 10, 11, 47]. Most of these studies were with mingled residual/persistent and recurrent OSCC cases, within whom a higher proportion of patients were found with histories of prior radiotherapy or chemoradiation [4, 31, 47]. We came up with the first report for the outcomes of immediate SS-based treatment against residual/persistent OSCCs, who were mostly radiation-naive. The answer of salvage likelihood for residual/persistent OSCCs was partially answered in our study, as the survival outcomes diversified among these patients. According to us, careful case selections for SS should be emphasized based on both the initial and residual status. In the current study, patients with both smaller primary and residual OSCC sizes were mostly salvageable under a sound retreatment. However, for cases with larger residual disease burdens, the prognosis was generally unfavorable with a meager survival of 15.4%. The involvement of vital structures in residual OSCCs were also shown to decrease the likelihood of rescue. Within these, the extremely unfavorable outcome of 3 cases with carotid involvement and sacrifices alarmed us a possible contraindication when oncologic evidence of internal carotid artery wrapping was found. As for the treatment designs, we found a slight advantage of survival for the SS group over the AT-SS group. A stronger association was also found for the salvage resection and reconstruction extent, as most patients with wide margin re-resections and free-flap (including PMMF) reconstructions enjoyed better survival outcomes. Adjuvant radiotherapy or chemoradiation following SS should be considered for patients with residual/persistent OSCCs, for a 10–20% survival advantage, reported in other studies [48, 49]. As for other treatment combinations, the effects of targeted (EGFR or VEGF-based) therapies fell short of expectations as the trends of treatment outcomes reversed despite such added treatment regimens. We owed this phenomenon to both the treatment toxicities, and to the more advanced disease status of those who were inclined to receive such treatment combinations. As far as we are concerned, routine postoperative radiotherapy or chemoradiation is able to reach a similar, or even better outcome without supplement of targeted therapies, judging from results in our statistics.

Undoubtedly, some limitations were inherent in the present study. Firstly, our results were obtained in a retrospective cohort in a single institution. Secondly, the treatment benefits for advanced residual cases were unable to summarize due to the small number in this investigation. Most patients were also irradiation-naïve in the primary treatment. In addition, the case selection for curative SS were quite subjective. Lastly, the effects of immunotherapies were elusive given the absence of such treatment at that time.

## Conclusions

To sum up, from our results, patients with residual/persistent OSCCs are still surgically salvageable with acceptable outcomes (OS: 60.2%). Among all the variables, we found strong associations between OS and primary T stage (*p* = 0.003), plus residual/persistent lesion size (*p* < 0.001), based on the final Cox multivariate analysis. Thus, to achieve optimal efficacies, it is quite necessary to select suitable cases with residual/persistent diseases for SS, as SS is no panacea for all OSCC cases. In our opinion, SS for cases with both smaller primary and residual OSCCs, especially those with radiation-naïve features is still feasible, when carefully designed and performed.

Lessons should be learned that when encountered with each primary OSCC case, a well-round, evidence-based surgical plan, together with an able surgical expertise, is mandatory for the ultimate treatment success. Cases with residual/persistent OSCCs were mostly due to mistakes which could have been avoided if the guidelines and practice codes were strictly followed. In the near future, to improve the quality of care, we strongly advocate a domestic virtual/online hub for telemedicine and case sharing, which facilitates learning among those from low-volume, resources-deprived areas, along with timely supervision by experts from high-volume academic health centers. Besides, initiatives should be taken to commencing online or hands-on training modules for updated knowledge of OSCC diagnosis and treatment guidelines, which are mandatory, rather than voluntary for those who are willing to continue providing OSCC treatment. The professional threshold and supervision should be strengthened with a domestic/regional committee assessing the outcomes of treatment on a regular schedule. For the sake of patients, accountability system is also advised to establish to confirm that appropriate diagnosis and treatment is offered without iatrogenic delay.

## Supplementary Information

**Additional file 1.** Supplementary Fig. 1. Representative Case 1. A female patient, aged 65, received surgical treatment for primary T3N0M0 right tongue squamous cell carcinoma 4 years ago in another institution. She was referred to our department for retreatment due to the postoperative reports revealing both inner and deep positive margins. SS and re-neck dissection were performed with reconstructive methods of the anterolateral thigh flap (ALTF). The residual tongue disease reached 3.8 cm according to the pathological report. The patient then received postoperative radiochemotherapies with an uneventful course for 37 months. A: The intraoral view of the resected tongue and residual tumor. B: PET-CT scan of the residual lesion in the deeper tongue region. C: The specimen after SS. D: The reconstructed right tongue.

**Additional file 2.** Representative Case 2. A 47 years old male patient received surgical treatment for tongue squamous cell carcinoma (T2N2M0) in another institution. The initial treatment included hemi-glossectomy with modified radical neck dissection in the ipsilateral side. Although the postoperative margin reports were negative, the patient was with persistent symptoms of pain and firmness in the ipsilateral neck right after the operations. Ulcerated mass was found 2 months after the operation and was referred to our hospital for re-treatment. Due to the involvement of nearby arterial structures, the carotid artery was ligated during the extensive resection of the residual lesions. The defect was covered with ALTF reconstructions. Despite postoperative radiotherapy, he developed local re-recurrence at 7 months, and died at 9 months during the follow-up. A: Intraoral view of tongue defect and radial forearm flap. B: The cervical ulcerated mass (residual tumors). C: The axial CT revealed the residual mass around the carotid artery. D: Intraoperative view of the residual tumor. E: An extensive resection with carotid artery ligation was performed for SS. F: The defect was covered with an ALTF.

**Additional file 3.** Representative Case 3. A 65 years old male patient was referred for the contralateral-side positive margin after surgeries for lower gingival cancer (T2N1M0). Postoperative radiotherapy was offered to him, but later ceased at 8 Gy due to the request of the patient. He came to our clinics for re-treatment. The mass was suspected on the contralateral side with firmness in the submental region. He then received SS resection of the mass and the defect was also covered with ALTF. The residual lesion reached 3.2 cm according to the pathological report. He then continued radiotherapy for 56 Gy and no adverse event was reported during the 49 months postoperative follow-up. A: The suspected mass in the contralateral submental region. B. The intraoperative view of the planned resection. C: The intraoperative view after residual tumor resection. D: The defect was covered with ALTF.

**Additional file 4.** Representative Case 4. A female patient aged 67 was referred to our institution for a retreatment of buccal cancer (T3N0M0) after surgical treatment elsewhere. The initial treatment was only directed for local excision without preoperative biopsy. The postoperative report confirmed the diagnoses of squamous cell carcinoma and found positive deep margin due to the initial conservative treatment. She refused retreatment in the previous institution and came to our hospital 6 weeks later with an enlarging mass in the left cheek. We performed a radical resection of the residual mass and the defect was reconstructed with ALTF. She then received sequential radio-chemotherapy and no adverse event was reported during the 29 months of follow-up. A: The residual mass in the left side of the cheek. B: Intraoperative view of the cheek mass. C: The specimen after SS. D: The defect was reconstructed with ALTF.

## Data Availability

The datasets used and/or analyzed during the current study are available from the corresponding author (Yue He) on reasonable request.

## Abbreviations

OSCC: oral squamous cell carcinoma
SS: salvage surgery
AJCC: American Joint Committee on Cancer
DOI: depth of invasion
ENE: extra-nodal extension
MiRDs: microscopic residual diseases
MaRDs: macroscopic residual diseases
RRDs: rapid recurrent (persistent) diseases
PMMF: pectoralis major myocutaneous flap
PD-1: programmed death-1
OS: overall survival
NCCN: National Comprehensive Cancer Network
AT-SS: adjuvant treatment regimens first, followed by SS
