# Supplementary figures and images for: Salvage surgery for patients with residual/persistent diseases after improper or insufficient treatment of oral squamous cell carcinoma: can we rectify these mistakes?
Source: BMC Cancer. 2021 Jul 31;21:878. doi: 10.1186/s12885-021-08600-2 (PMC8325844; doi:10.1186/s12885-021-08600-2)

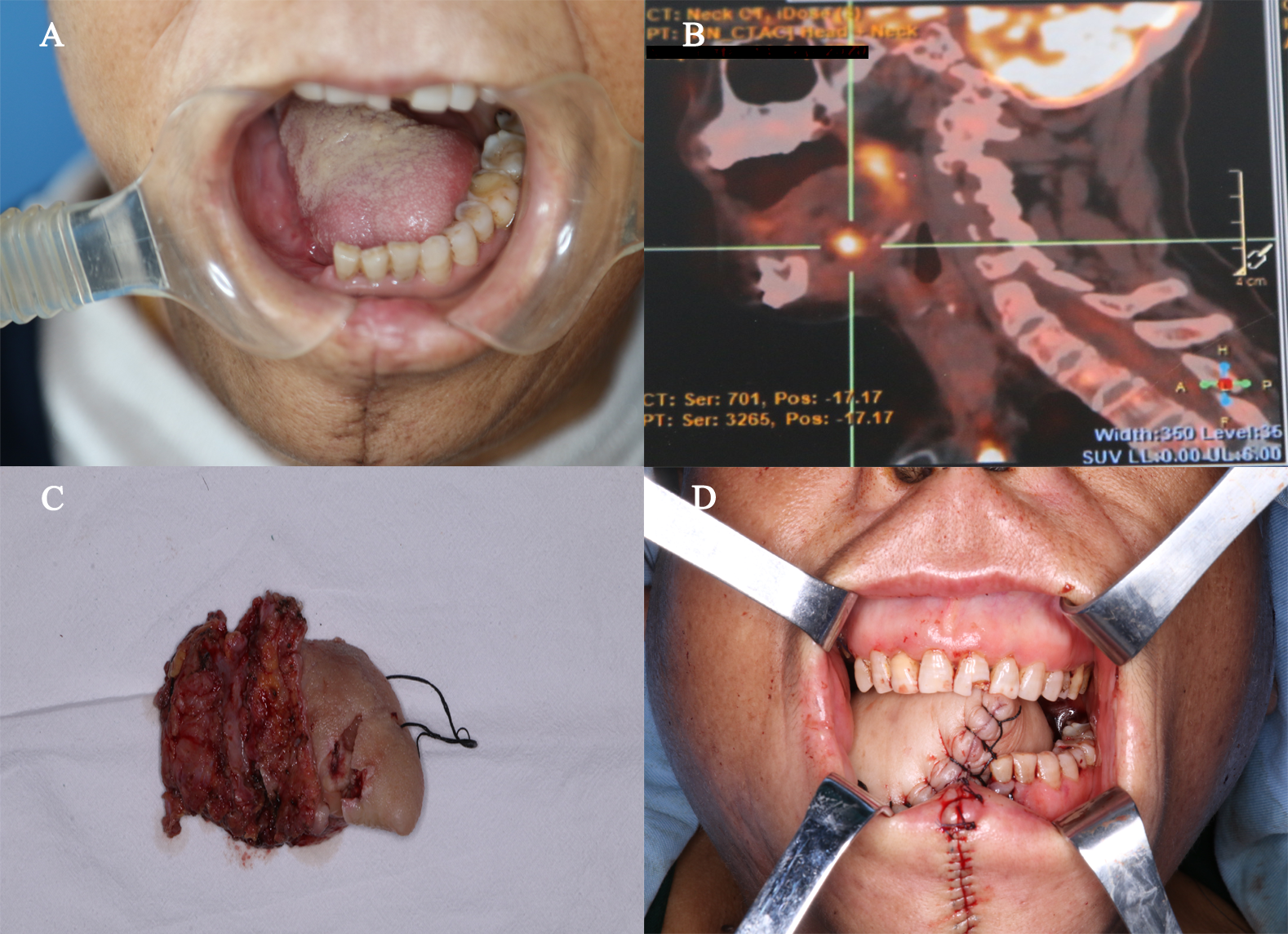

Supplement: Supplementary file 1 — Additional file 1. Supplementary Fig. 1. Representative Case 1. A female patient, aged 65, received surgical treatment for primary T3N0M0 right tongue squamous cell carcinoma 4 years ago in another institution. She was referred to our department for retreatment due to the postoperative reports revealing both inner and deep positive margins. SS and re-neck dissection were performed with reconstructive methods of the anterolateral thigh flap (ALTF). The residual tongue disease reached 3.8 cm according to the pathological report. The patient then received postoperative radiochemotherapies with an uneventful course for 37 months. A: The intraoral view of the resected tongue and residual tumor. B: PET-CT scan of the residual lesion in the deeper tongue region. C: The specimen after SS. D: The reconstructed right tongue. [file 12885_2021_8600_MOESM1_ESM.tif]

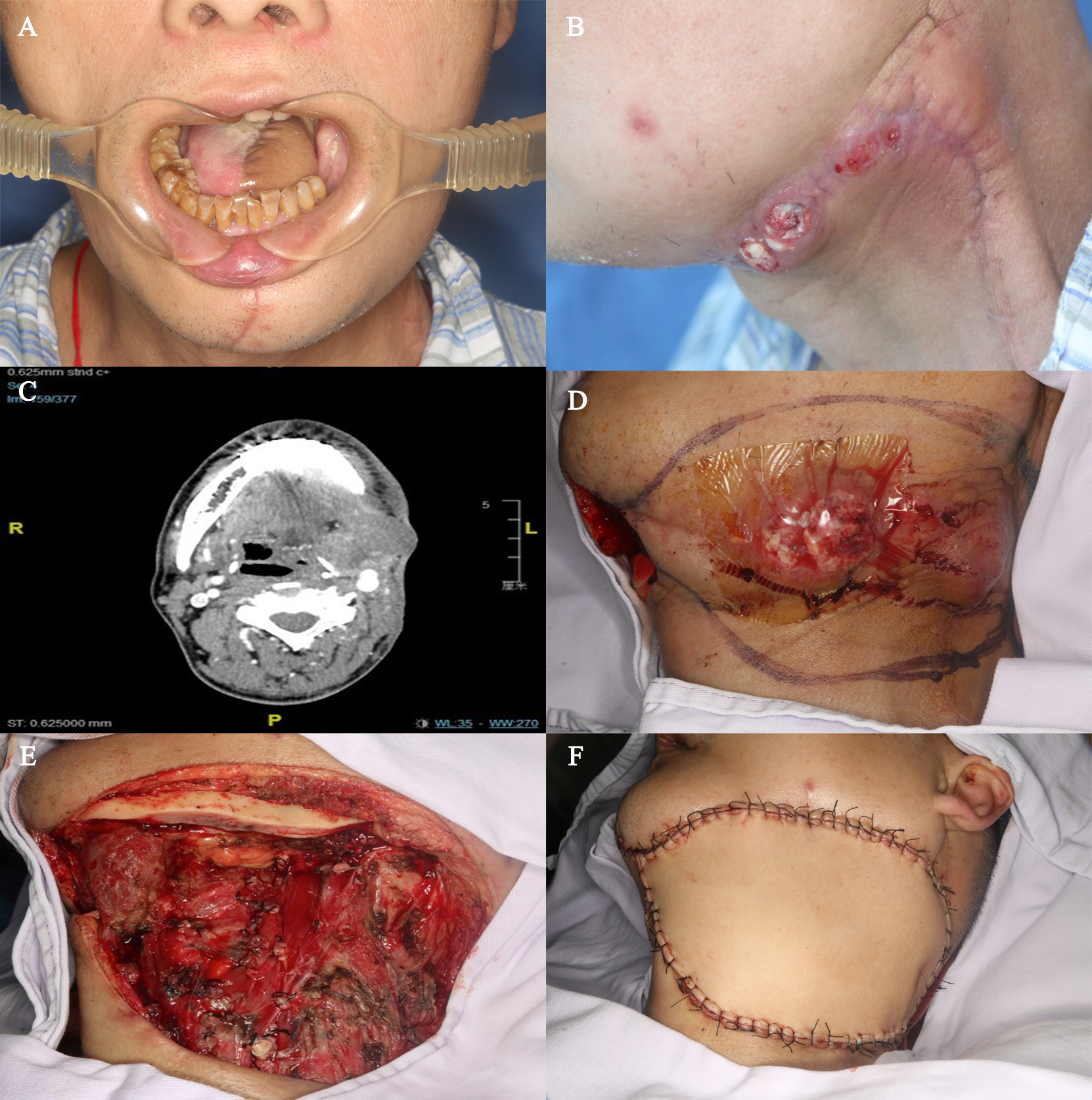

Supplement: Supplementary file 2 — Additional file 2. Representative Case 2. A 47 years old male patient received surgical treatment for tongue squamous cell carcinoma (T2N2M0) in another institution. The initial treatment included hemi-glossectomy with modified radical neck dissection in the ipsilateral side. Although the postoperative margin reports were negative, the patient was with persistent symptoms of pain and firmness in the ipsilateral neck right after the operations. Ulcerated mass was found 2 months after the operation and was referred to our hospital for re-treatment. Due to the involvement of nearby arterial structures, the carotid artery was ligated during the extensive resection of the residual lesions. The defect was covered with ALTF reconstructions. Despite postoperative radiotherapy, he developed local re-recurrence at 7 months, and died at 9 months during the follow-up. A: Intraoral view of tongue defect and radial forearm flap. B: The cervical ulcerated mass (residual tumors). C: The axial CT revealed the residual mass around the carotid artery. D: Intraoperative view of the residual tumor. E: An extensive resection with carotid artery ligation was performed for SS. F: The defect was covered with an ALTF. [file 12885_2021_8600_MOESM2_ESM.tif]

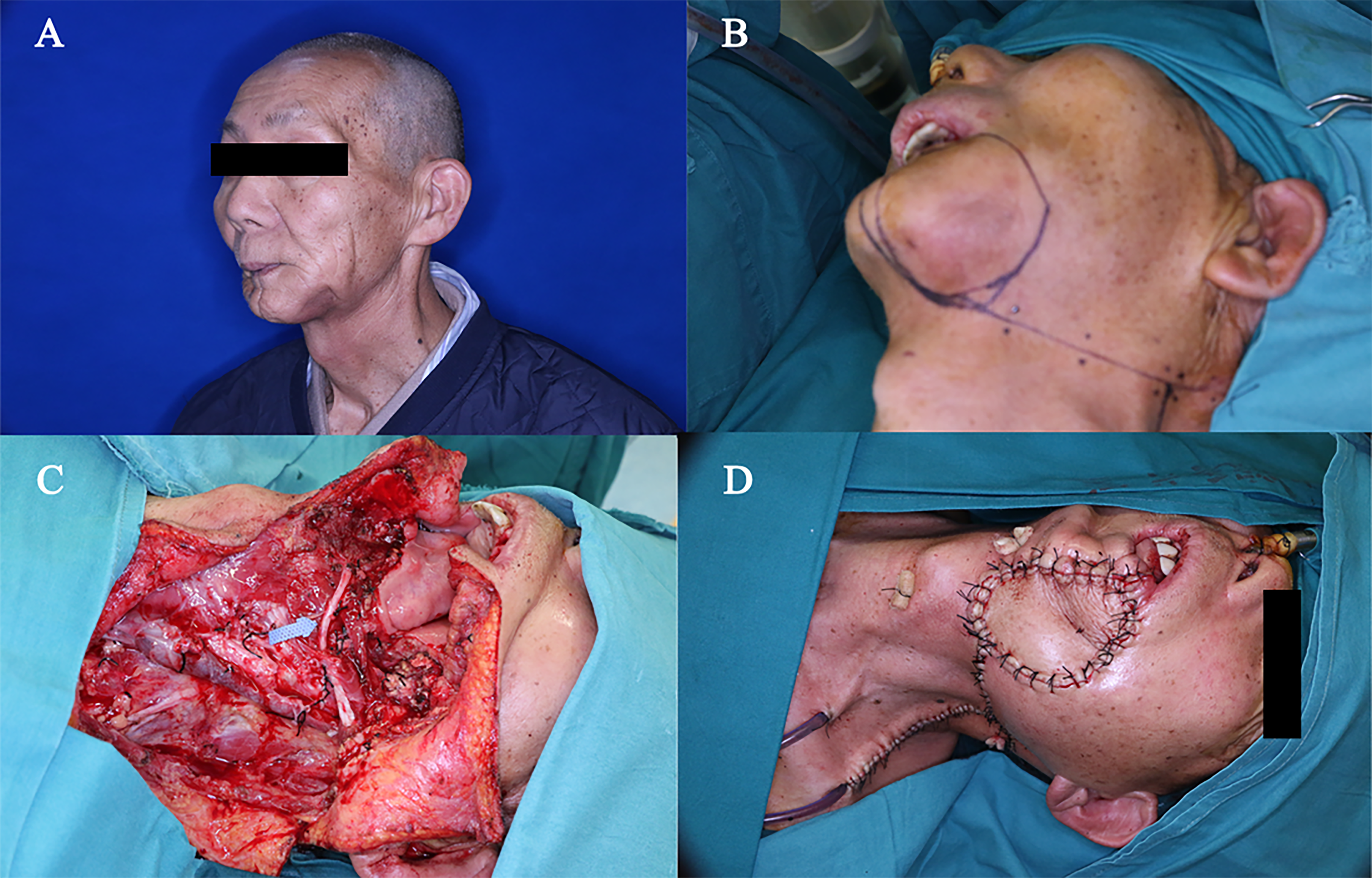

Supplement: Supplementary file 3 — Additional file 3. Representative Case 3. A 65 years old male patient was referred for the contralateral-side positive margin after surgeries for lower gingival cancer (T2N1M0). Postoperative radiotherapy was offered to him, but later ceased at 8 Gy due to the request of the patient. He came to our clinics for re-treatment. The mass was suspected on the contralateral side with firmness in the submental region. He then received SS resection of the mass and the defect was also covered with ALTF. The residual lesion reached 3.2 cm according to the pathological report. He then continued radiotherapy for 56 Gy and no adverse event was reported during the 49 months postoperative follow-up. A: The suspected mass in the contralateral submental region. B. The intraoperative view of the planned resection. C: The intraoperative view after residual tumor resection. D: The defect was covered with ALTF. [file 12885_2021_8600_MOESM3_ESM.tif]

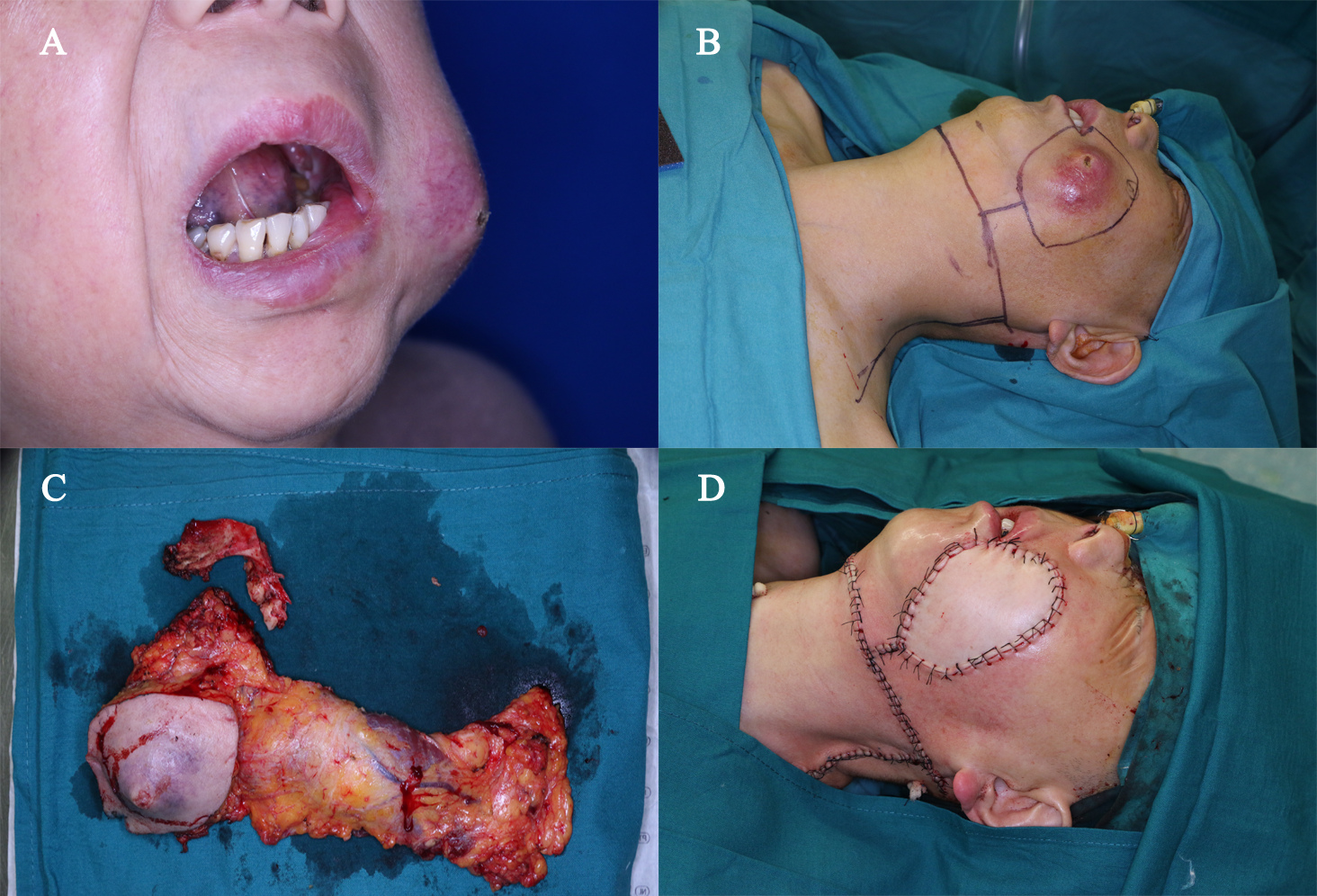

Supplement: Supplementary file 4 — Additional file 4. Representative Case 4. A female patient aged 67 was referred to our institution for a retreatment of buccal cancer (T3N0M0) after surgical treatment elsewhere. The initial treatment was only directed for local excision without preoperative biopsy. The postoperative report confirmed the diagnoses of squamous cell carcinoma and found positive deep margin due to the initial conservative treatment. She refused retreatment in the previous institution and came to our hospital 6 weeks later with an enlarging mass in the left cheek. We performed a radical resection of the residual mass and the defect was reconstructed with ALTF. She then received sequential radio-chemotherapy and no adverse event was reported during the 29 months of follow-up. A: The residual mass in the left side of the cheek. B: Intraoperative view of the cheek mass. C: The specimen after SS. D: The defect was reconstructed with ALTF. [file 12885_2021_8600_MOESM4_ESM.tif]
